# Next-generation neoantigen mRNA vaccines: Immuno-engineering strategies for precision cancer immunotherapy

**DOI:** 10.1007/s13402-026-01199-1

**Published:** 2026-04-15

**Authors:** Xiaoping Li, Parham Jabbarzadeh Kaboli, Ghazaal Roozitalab, Shanli Salahi, Hongbo Qian, Xinyi Zhang, Keda Chen, Saber Imani

**Affiliations:** 1 Key Laboratory of Artificial Organs and Computational Medicine in Zhejiang Province, Shulan International Medical College, Zhejiang Shuren University, Hangzhou, Zhejiang China; 2 Research & Innovation Lab, OncoVanguard Media, Texas Medical Center, Houston, TX 77025 USA; 3 Department of Metallurgical and Materials Engineering, Faculty of Technology, University of Gazi, Ankara, 06500 Turkey; 4 School of Health in Social Science, University of Edinburgh, Edinburgh, UK; 5 Shulan International Medical College, Zhejiang Shuren University, Hangzhou, Zhejiang China

**Keywords:** Neoantigen mRNA vaccines, Immunoengineering, Translational manufacturing, Thermostable formulations, Biomaterials-based delivery, Antigen presentation engineering

## Abstract

Neoantigen mRNA vaccines have progressed from experimental constructs to clinically evaluated immunotherapies grounded in tumor genomics and modular RNA engineering. By encoding tumor-restricted antigens, these platforms aim to induce targeted T-cell responses while minimizing off-target toxicity. Early clinical studies, particularly in melanoma and non-small cell lung cancer, demonstrate that personalized and hybrid vaccine strategies can expand tumor-reactive T-cell clones and improve recurrence-free outcomes when combined with immune checkpoint blockade. However, challenges including manufacturing timelines, HLA diversity, tumor heterogeneity, and immune editing limit broad implementation. Recent advances in antigen prioritization algorithms, transcript design, and delivery platforms have improved translational feasibility and reduced production variability. Shared and off-the-shelf approaches targeting recurrent driver mutations offer scalable alternatives, while adaptive strategies incorporating ctDNA monitoring raise the possibility of dynamic vaccine updating during treatment. Integration with immune-modulating therapies and rational clinical positioning, particularly in adjuvant and minimal residual disease settings, are likely to define near-term success. Collectively, neoantigen mRNA vaccination represents a flexible therapeutic framework rather than a single platform. Its long-term impact will depend on aligning molecular engineering, immune calibration, manufacturing scalability, and regulatory adaptation to achieve durable clinical.

## Introduction

Neoantigen mRNA vaccines have rapidly transitioned from experimental platforms to clinically promising immunotherapeutics, redefining individualized neoantigen therapy (INT) [1]. By encoding tumor-specific mutations, these vaccines direct cytotoxic T-cell responses against antigens absent from normal tissues, representing a major advance in precision oncology. Although early clinical trials have demonstrated encouraging immunogenicity and preliminary efficacy, fully personalized vaccines remain limited by manufacturing timelines (6–8 weeks), high costs, and tumor heterogeneity [2–4].

mRNA vaccine design is guided by three principal antigen categories: tumor-specific antigens (TSAs), tumor-associated antigens (TAAs), and neoantigens [5]. TSAs arise from tumor-exclusive genomic alterations that generate non-self-peptides capable of eliciting potent T-cell responses with minimal central tolerance [6]. TAAs are self-derived proteins aberrantly expressed or overexpressed in malignant cells; while subject to immune tolerance, their recurrence across patients enables broader therapeutic applicability [7, 8]. Neoantigens, commonly viewed as a functional subset of TSAs, originate from nonsynonymous somatic mutations that form novel peptide–MHC complexes and underpin the conceptual framework of INT [9, 10].

These biological distinctions have led to four major mRNA vaccine strategies: personalized TSA-based vaccines, TAA-based platforms, hybrid constructs integrating both [11], and shared neoantigen vaccines targeting recurrent oncogenic mutations for off-the-shelf use [12]. Each approach reflects a trade-off between precision and scalability.

Advances in epitope prediction algorithms, next-generation sequencing, and immunopeptidomics have accelerated antigen discovery [13]. Concurrent improvements in mRNA engineering—including nucleotide modification, untranslated region optimization, and lipid nanoparticle (LNP) delivery—have enhanced transcript stability and immune activation [13]. Emerging thermostable and spray-dried formulations further address distribution and storage constraints [14]. Together, these innovations have improved T-cell priming across diverse HLA backgrounds and partially mitigated immune escape and inter-patient variability [15].

This review examines the evolution of neoantigen mRNA vaccines from individualized constructs to scalable TSA, TAA, hybrid, and shared platforms. We integrate advances in antigen discovery, mRNA design, delivery systems, and translational development to clarify the clinical and mechanistic rationale guiding next-generation neoantigen immunotherapy.

## Technological milestones in neoantigen mRNA vaccine development

**Fig. 1:**
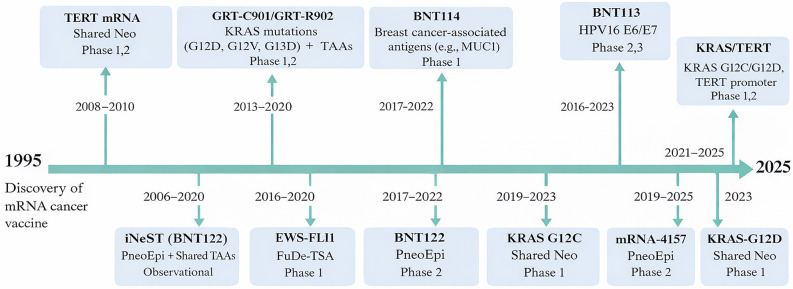
Milestones in neoantigen mRNA vaccine development (1995–2025). Timeline highlighting the transition from early discovery to clinical trials targeting personalized and shared tumor antigens using mRNA vaccine platforms. Advances in sequencing, antigen discovery, and mRNA engineering have enabled rapid clinical translation of neoantigen vaccines

Prior to 2012, mRNA cancer vaccine research was largely confined to preclinical studies. Early murine experiments demonstrated that direct administration of mRNA encoding tumor antigens could induce antigen-specific cytotoxic T lymphocyte (CTL) responses. However, clinical translation was limited by intrinsic RNA instability, excessive innate immune activation, inefficient in vivo translation, and the absence of reliable delivery systems. In parallel, the field lacked standardized pipelines for neoantigen identification and validation, further restricting clinical scalability [3, 16] (Fig. 1). 

A major inflection point occurred in 2012 with the introduction of chemically optimized mRNA constructs designed to enhance transcript stability and translational efficiency while limiting unwanted innate immune sensing. These advances improved dendritic cell uptake and enabled systemic administration, forming the technological foundation for clinical-grade mRNA vaccines [16]. Between 2014 and 2015, the integration of whole-exome sequencing (WES), RNA sequencing, and immunopeptidomics transformed antigen discovery. Combined with rapidly evolving HLA-binding prediction algorithms and immunogenicity scoring tools, these platforms enabled systematic identification of patient-specific mutations suitable for INT [17]. Importantly, parallel improvements in custom synthesis workflows made early-phase clinical testing feasible.

From 2016 onward, early clinical studies (e.g., NCT03468244, NCT03908671) demonstrated that personalized mRNA vaccines could induce measurable CD8⁺ and CD4⁺ T-cell responses in patients [15, 18]. Yet practical constraints quickly emerged. Manufacturing timelines frequently exceeded six weeks, coordination between sequencing, bioinformatics, and production was complex, and tumor evolution during this interval could alter antigen landscapes. These limitations catalyzed a strategic shift toward semi-personalized and shared platforms.

Subsequent development efforts focused on recurrent TAAs and shared neoantigens derived from common oncogenic mutations such as KRAS and TERT, as well as fusion-derived TSAs including BCR-ABL and EWS-FLI1 [9, 19–21]. These targets enable more standardized manufacturing, reduce production latency, and facilitate off-the-shelf deployment. Such platforms are particularly compatible with immune checkpoint blockade and cytokine-based combination regimens, broadening their clinical applicability [22–24].

Concurrently, advances in LNP technology, thermostable and lyophilized formulations, and alternative administration routes (including mucosal delivery) have improved biodistribution, storage flexibility, and global accessibility [25]. Large-scale clinical initiatives—including population-level personalized vaccine programs and multi-tumor mRNA vaccine trials—reflect increasing regulatory confidence and industrial maturation of the field [26]. The recent FDA clearance of universal tumor-targeting mRNA formulations further underscores the transition from proof-of-concept studies to structured translational pipelines [27].

Together, these milestones illustrate the evolution of neoantigen mRNA vaccines from experimental constructs constrained by instability and limited targeting tools to integrated, clinically oriented platforms supported by advanced sequencing, predictive immunology, and scalable manufacturing infrastructure (Fig. 1).

## Alternative neo-antigen mRNA vaccine strategies

Although fully INT platforms represent the most precise implementation of mRNA cancer vaccination, their clinical translation remains technically demanding and resource-intensive [28–32]. In parallel, TAA-based platforms leverage antigens that are consistently overexpressed in defined tumor types. While these constructs face tolerance-related constraints, their recurrence enables broader patient coverage and streamlined production workflows [9, 10, 31].

**Fig. 2:**
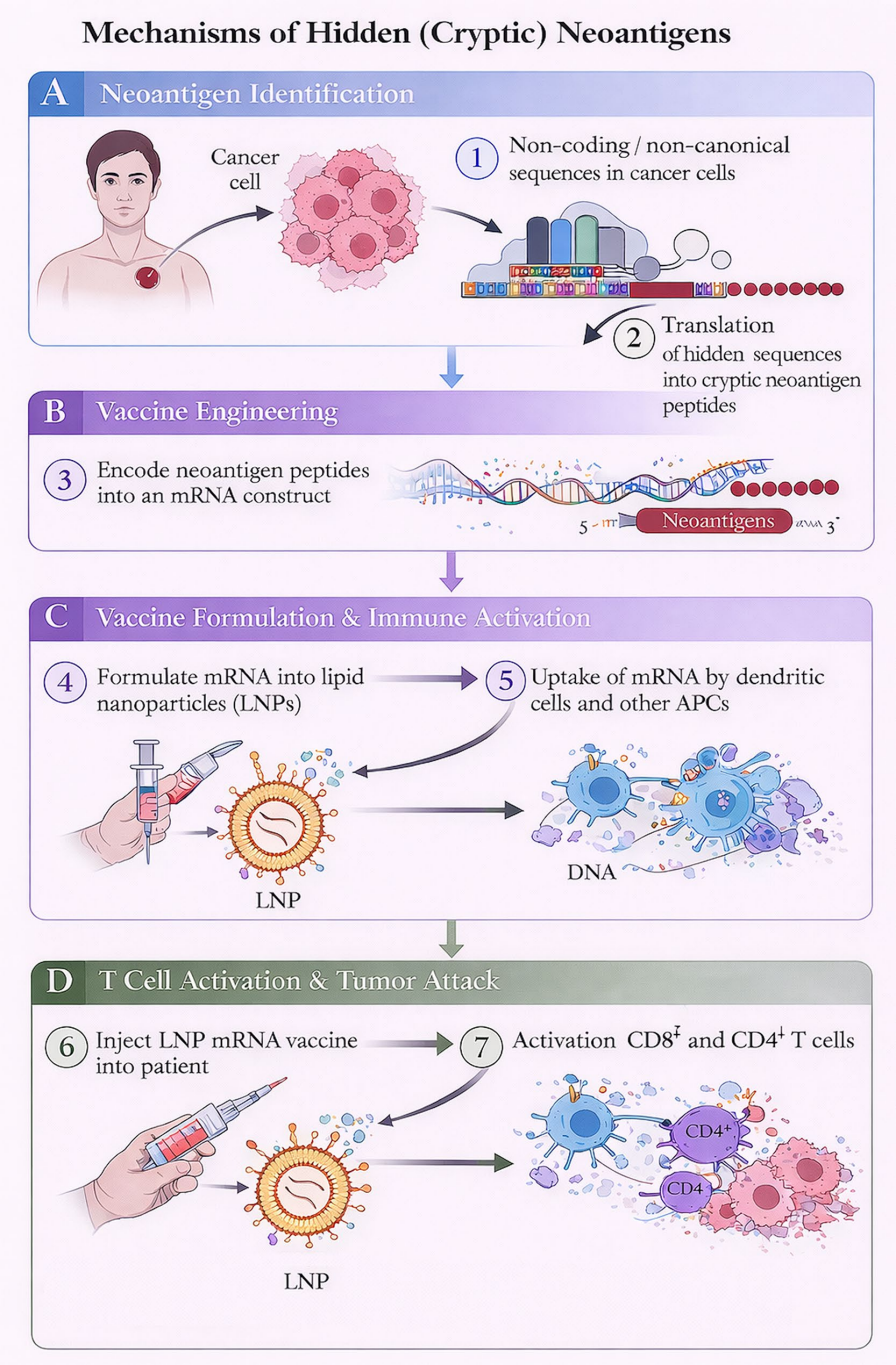
Design strategies for personalized versus shared neoantigen mRNA vaccines. This schematic compares two major approaches for mRNA cancer vaccine development. **(A)** Patient-specific vaccines are generated through tumor sequencing to identify unique neoepitopes, enabling individualized mRNA constructs (e.g., BNT122, mRNA-4157) that induce targeted immune responses against tumor-specific mutations. **(B)** In contrast, shared antigen vaccines encode recurrent tumor antigens present across multiple patients (e.g., melanoma-associated antigens in BNT111), enabling off-the-shelf immunotherapy strategies. Personalized neoantigen vaccines maximize tumor specificity, whereas shared antigen vaccines offer scalable, broadly applicable immunotherapies

Hybrid vaccine designs integrate patient-specific mutations with shared TAAs within a single construct. This strategy aims to combine the high specificity of individualized targets with the manufacturing feasibility and antigen breadth of shared platforms. By increasing epitope diversity, hybrid approaches may also mitigate immune escape driven by antigen loss or clonal evolution [33, 34]. A further evolution involves off-the-shelf mRNA vaccines directed against common driver mutations or frequently expressed tumor antigens [35–37]. These platforms reduce production latency, facilitate batch manufacturing, and enable rapid integration with immune checkpoint inhibitors or cytokine-based combinations. Compared with fully individualized approaches, off-the-shelf constructs prioritize deployment speed and population-level applicability over maximal personalization [4] (Fig. 2).

Collectively, these strategies reflect a spectrum rather than discrete categories, balancing precision, scalability, and clinical practicality. Ongoing trials evaluating these platforms across multiple malignancies are summarized in Table 1, which details antigen class, vaccine architecture, and clinical context under investigation.

**Table 1:** Overview of representative mRNA vaccine candidates targeting tumor-specific and tumor-associated antigens across different cancer types. The table summarizes ongoing and completed clinical studies investigating personalized, shared, hybrid, and off-the-shelf mRNA vaccine strategies

Trials	Vaccine	Antigen Type	Targets	Indication	Phase	Primary Outcome	Ref.
NCT04526899	BNT111	TAAs	MAGE-A3, NY-ESO-1, Tyrosinase, TPTE	Advanced Melanoma	II	Immune	[34]
NCT04534205	BNT113	TAAs	HPV16 E6/E7	HPV16 + HNSCC	II, III	Immune / Clinical	[9, 10]
-	BNT114	TAAs	MUC1 and other breast tumor-associated antigens	TNBC	Preclinical	Immune	[38]
-	Cryptic NeoAgs	Fusion TSA	nc ORFs, lncRNA-derived peptides	GBM, NSCLC, PDAC	Preclinical	Immune	[24]
NCT02736565	EWS-FLI1	Fusion TSA	EWS-FLI1 fusion peptide	Ewing Sarcoma	I	Immune	[9, 19]
NCT05993455	KRAS G12C	Shared Neo	KRAS G12C	NSCLC, CRC, PDAC	II	Immune	[21]
NCT06218914	KRAS G12D	Shared Neo	KRAS G12D	Metastatic PDAC	I	Immune	-
NCT05726864	KRAS/TERT	Off-the-Shelf	KRAS G12C/G12D, TERT promoter mutation	Multiple cancers (e.g., GBM, NSCLC, PDAC)	I, II	Immune	[20]
NCT04161755	BNT122^*^	TSAs	PneoEpi	PDAC	I	Immune / Clinical	[32]
NCT01363596	iNeST (BNT122)^**^	Hybrid^***^	PneoEpi + Shared Neo	Melanoma	I	Immune	[33]
NCT03897881	mRNA-4157	TSAs	PneoEpi	Stage III/IV Melanoma	IIb	Immune	-
NCT03639714	GRT-C901/GRT-R902	Shared Neo + Off-the-Shelf	KRAS mutations (G12D, G12V, G13D) + TAAs	Metastatic solid tumors (CRC, NSCLC, PDAC)	Phase I/II	Immune / Molecular	[36, 37]

^*^ BNT122, also known as autogene cevumeran, is a personalized mRNA neoantigen vaccine evaluated in patients with resected pancreatic ductal adenocarcinoma (PDAC) and other solid tumors.^**^ BNT122 is being investigated within the iNeST (individualized neoantigen-specific immunotherapy) platform, which encodes patient-specific neoepitopes and may incorporate additional shared tumor antigens depending on the vaccine design and tumor context.^***^ Hybrid neoantigen vaccine strategies integrate tumor-specific antigens (TSAs) and tumor-associated antigens (TAAs) within a single mRNA construct to enhance immunogenicity while preserving elements of tumor specificity.**Abbreviations**: PneoEpi, personalized neoepitopes; TAA, tumor-associated antigen; TSA, tumor-specific antigen; HNSCC, head and neck squamous cell carcinoma; GBM, glioblastoma multiforme; NSCLC, non–small cell lung cancer; CRC, colorectal cancer; mPanCA, metastatic pancreatic cancer; PDAC, pancreatic ductal adenocarcinoma; TNBC, triple-negative breast cancer; ncORFs, non-canonical open reading frames; lncRNAs, long non-coding RNAs.

### Tumor-specific antigens mRNA vaccination

TSAs represent a biologically privileged class of neoantigens that arise exclusively in malignant cells and are absent from normal tissues. Their tumor-restricted expression enables recognition as non-self, permitting high-avidity CTL responses without the central tolerance constraints that frequently limit TAA-directed immunity [39]. These antigens originate from diverse genomic alterations, including SNVs, indels, gene fusions, intron retention, and aberrant splicing, which generate novel peptides presented on HLA moleculesand targeted by T cells [6]. Immunopeptidomic analyses have further expanded this landscape by identifying cryptic peptides derived from non-canonical translation events, alternative reading frames, and unconventional transcripts [32, 40–42]. Such peptides are efficiently presented on tumor HLA molecules and recognized by tumor-infiltrating lymphocytes [43], and accumulating evidence suggests that cryptic antigens may be as abundant as mutation-derived TSAs in certain malignancies [44, 45]. This expanded repertoire is particularly relevant in tumors with low mutational burden, where classical mutation-derived targets may be limited.

The identification of TSAs has been accelerated by advances in NGS and computational pipelines enabling matched tumor–normal analyses. Predictive tools such as dbPepNeo [46], NetMHCpan [47], and MHCflurry [48] prioritize candidate epitopesbased on peptide–HLA binding affinity, and recently developed frameworks optimized for direct tumor–normal sequence comparison improve precision in identifying tumor-exclusive neoepitopes [49]. However, in silico prediction remains imperfect, frequently generating false positives that require validation using patient-derived T cells [50]. This validation step is not merely technical but clinically essential, as off-target recognition of self-antigens has been associated with severe immune-related toxicity in prior clinical experiences [51]. Accurate antigen prioritization therefore remains central to both efficacy and safety.

Clinical translation of TSA-based mRNA vaccines has demonstrated that these biological principles can be operationalized. Autogene cevumeran (BNT122) evaluated in resected pancreatic ductal adenocarcinoma induced de novo neoantigen-specific T-cell responses in approximately half of treated patients, and responders exhibited prolonged recurrence-free survival relative to non-responders [32, 33]. These findings suggest that TSA vaccination can introduce immunogenic epitopes into otherwise poorly infiltrated tumors and may sensitize immunologically “cold” malignancies to subsequent immune modulation (Table 1; Fig. 2).

**Fig. 3:**
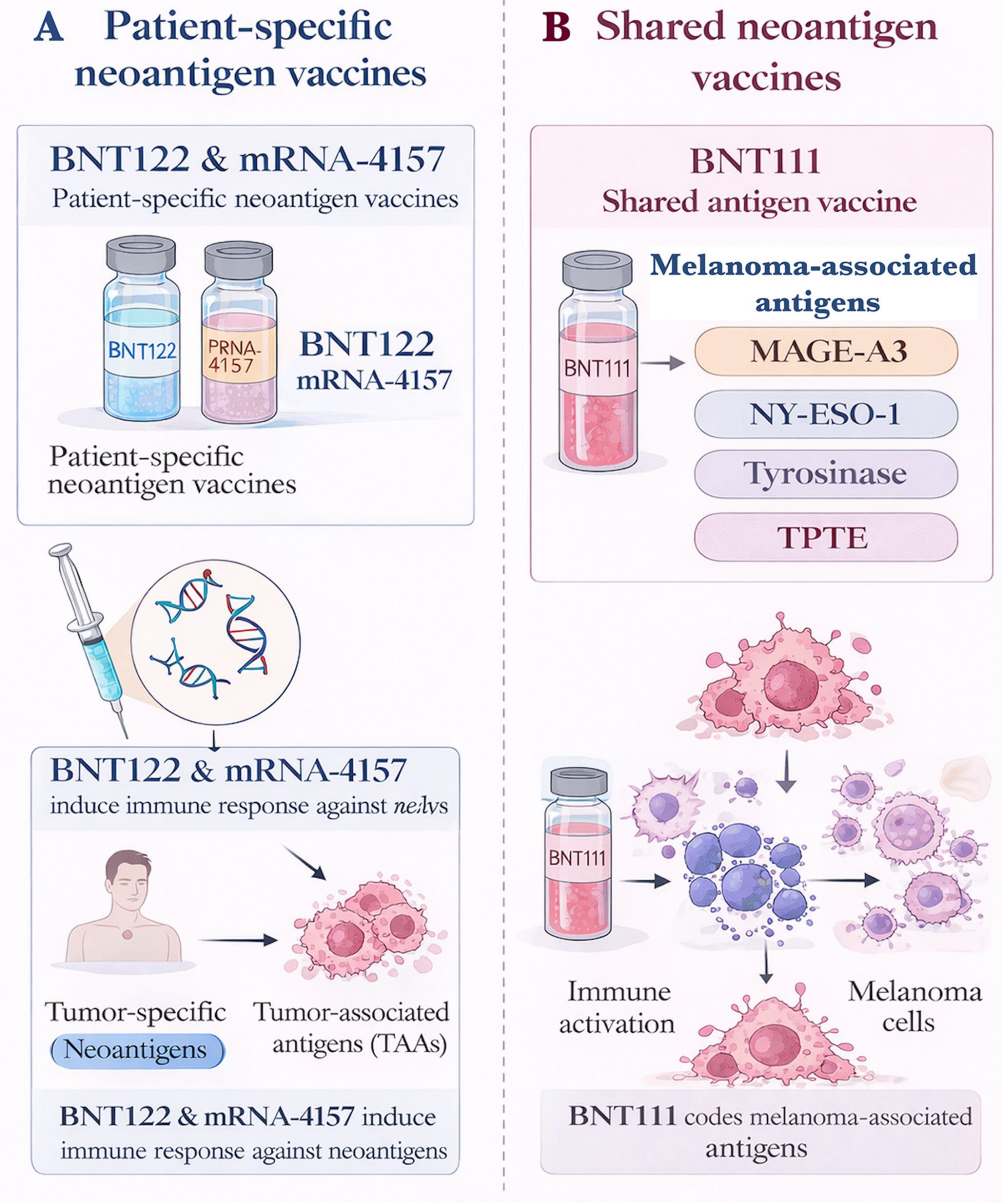
Cryptic neoantigens as emerging targets for mRNA cancer vaccines. This schematic illustrates the generation and therapeutic exploitation of cryptic neoantigens derived from non-canonical open reading frames (ORF) and non-coding genomic regions in cancer cells. Aberrant transcription or translation events, such as alternative splicing or intron retention, produce novel peptides that are absent in normal tissues. These cryptic peptides can be encoded in mRNA vaccines, delivered via lipid nanoparticles, and presented by antigen-presenting cells to activate CD4⁺ and CD8⁺ T-cell responses against tumor cells. Cryptic neoantigens expand the pool of tumor-specific targets, offering new opportunities to overcome immune escape and improve precision cancer immunotherapy

Despite this promise, both logistical and biological challenges persist. Personalized workflows—from tumor sampling through sequencing, antigen prioritization, mRNA synthesis, LNP encapsulation, and GMP release—require several weeks, limiting applicability in rapidly progressing disease [52]. In addition, immune pressure may select for antigen loss, particularly when vaccines target subclonal mutations. Multi-epitope designs incorporating both clonal and subclonal targets have therefore been proposed to enhance response durability and mitigate immune escape [53]. The vaccinable landscape continues to expand through identification of fusion-driven TSAs such as EWS-FLI1 and BCR-ABL, as well as non-canonical sources including upstream open reading frames, endogenous retroelements, intronic regions, and lncRNA transcripts [6, 19, 54, 55] (Fig. 3). Oncogenic pathway alterations, including PIK3CA mutations in TNBC, may further reshape tumor antigenicity and generate shared tumor-restricted epitopes suitable for semi-universal incorporation [56–59], paralleling observations in BRAF-driven melanoma [33, 57].

Looking forward, modular vaccine architectures integrating patient-specific and shared TSAs aim to balance personalization with manufacturability. Combination strategies with checkpoint inhibitors, type I interferon agonists, and agents targeting immunosuppressive pathways such as A2AR and TGF-β signaling are increasingly pursued to enhance efficacy and prevent immune escape [60]. Real-time ctDNA monitoring offers an additional layer of adaptability, enabling dynamic tracking of clonal evolution and potential refinement of vaccine composition during therapy [61]. As antigen prioritization, manufacturing workflows, and combination paradigms continue to mature, TSA-based mRNA vaccines are likely to find their most immediate impact in adjuvant and minimal residual disease settings, where tumor burden is limited and immune pressure can be strategically leveraged [62].

### Tumor-associated antigens mRNA vaccination

TAAs represent a distinct immunologic category characterized by aberrant expression, overexpression, mutation, or altered post-translational modification in malignant cells, with limited expression in normal adult tissues. Unlike TSAs, which are strictly tumor-restricted, TAAs are self-derived proteins and therefore exist within the spectrum of peripheral tolerance. Nevertheless, their recurrent expression across tumor types enables broader clinical applicability and more standardized manufacturing strategies. Frequently studied TAAs include CEA, HER2, WT1, and MUC1, all of which are widely expressed in solid tumors and hematologic malignancies [63]. Tumor-specific glycosylation changes, such as the hypoglycosylated form of MUC1 observed in adenocarcinomas, expose normally concealed peptide cores and create immunologically targetable epitopes [2, 64, 65]. Similarly, WT1 is overexpressed in glioblastoma and leukemia while remaining minimally expressed in most healthy tissues, supporting its development as a vaccine target [66, 67].

Clinical translation of TAA-based mRNA vaccination has demonstrated that shared antigen platforms can generate measurable immune responses. BNT111, encoding multiple melanoma-associated TAAs, showed early clinical activity in advanced melanoma, including in patients previously resistant to standard therapies [9]. Preclinical studies combining tumor-derived vesicle platforms with immune checkpoint blockade have further illustrated the capacity of TAA-directed vaccination to enhance cytokine production and T-cell activation in murine models [37]. These data underscore the potential of shared antigen constructs to amplify antitumor immunity when integrated into combination regimens.

Hybrid strategies further extend this concept. The mRNA-4157 platform integrates patient-specific neoantigens with shared TAAs, thereby balancing personalization with manufacturability. In the KEYNOTE-942 trial, combination therapy with pembrolizumab significantly improved recurrence-free survival and reduced distant metastasis risk in patients with high-risk resected melanoma, with a favorable safety profile [68]. These findings suggest that TAA-containing constructs may enhance immune breadth and durability when administered alongside checkpoint inhibition.

Despite these advances, TAA-directed vaccines face inherent biological challenges. Because TAAs are derived from self-proteins, immunodominance hierarchies and tolerance mechanisms can constrain response magnitude. Antigen loss, reduced epitope presentation, and defects in antigen-processing pathways further contribute to immune escape [69, 70]. Consequently, multi-epitope formulations and rational epitope prioritization are increasingly employed to broaden immune targeting and reduce vulnerability to single-antigen loss. Combination strategies with checkpoint blockade have shown preclinical efficacy, including restoration of CD8⁺ T-cell function in WT1-targeted models of leukemia [67]. In parallel, optimized LNP platforms, including PEGylated and pH-responsive formulations, enhance dendritic cell transfection and antigen expression, improving immunogenicity in vivo [71].

Table 1 summarizes ongoing clinical trials evaluating TAA-based mRNA vaccines across multiple malignancies, detailing antigen targets, construct design, and clinical stage. Collectively, TAA strategies occupy an intermediate position between individualized TSA platforms and fully shared neoantigen constructs, prioritizing scalability and antigen recurrence while requiring careful integration with immune-modulating therapies to overcome tolerance-related constraints.

### Shared neoantigen mRNA vaccination

Shared neoantigens arise from recurrent somatic driver mutations that are absent from the normal genome yet conserved across subsets of patients. Unlike TAAs, they remain tumor-specific and avoid central tolerance, while differing from fully individualized TSAs in their cross-patient recurrence. This dual property—specificity combined with scalability—positions shared neoantigens as strong candidates for off-the-shelf mRNA vaccine development [72].

Many shared neoantigens originate from oncogenic hotspot mutations. KRAS^G12C/D^ variants, frequently observed in pancreatic, colorectal, and non-small cell lung cancers, generate immunogenic epitopes presented by defined HLA alleles such as HLA-A*11:01 and HLA*-*C*08:02 [73, 74]. Similarly, recurrent TERT promoter mutations produce neoepitopes detected across glioblastoma, melanoma, and bladder cancer, supporting broader population-level targeting [75, 76]. Because these alterations often occur early in tumorigenesis and are clonally maintained, they may reduce intratumoral heterogeneity and mitigate the risk of antigen loss that complicates subclonal TSA strategies [77].

Preclinical data support the immunogenicity of this approach. mRNA constructs encoding KRAS^G12D^ have elicited robust CD8⁺ T-cell responses and tumor regression in murine models, prompting early-phase clinical evaluation of KRAS-targeted vaccines in metastatic pancreatic cancer [78]. These findings illustrate the translational logic of shared neoantigen vaccination: by focusing on conserved driver mutations, vaccine design can shift from bespoke manufacturing to population-stratified deployment.

However, identification and prioritization of shared neoantigens remain technically demanding. Predictive algorithms such as NetMHCpan and MHCflurry frequently overestimate peptide–HLA binding affinity, necessitating ex vivo validation to confirm true immunogenicity [46–48, 50]. Tumors may also evade immune pressure through antigen editing or pathway adaptation, reinforcing the importance of multi-epitope constructs targeting several recurrent mutations simultaneously. Emerging strategies therefore emphasize modular vaccine libraries incorporating common driver mutations across tumor types, although most remain in preclinical or early clinical stages [33, 79].

Beyond point mutations, fusion-derived neoantigens such as BCR-ABL and recurrent splicing-derived epitopes represent additional shared targets with strict tumor specificity [42, 80]. Preclinical evidence supports their immunogenicity and potential integration into semi-universal vaccine platforms [4]. Collectively, shared neoantigen vaccination represents a strategic midpoint between individualized and broadly shared antigen approaches, prioritizing clonal stability and manufacturability while preserving tumor specificity.

### Hybrid neo-antigen mRNA vaccination

Hybrid mRNA vaccine platforms seek to integrate individualized TSAs with recurrent TAAs or shared neoantigens within a single construct, thereby combining precision with scalability. Rather than positioning antigen categories as mutually exclusive, this strategy recognizes that tumor immunity may benefit from simultaneous targeting of clonal, subclonal, and shared determinants. By broadening antigenic coverage, hybrid constructs aim to enhance immune breadth, reduce the likelihood of antigen escape, and accommodate inter-patient heterogeneity.

Clinical translation of this concept has demonstrated feasibility. Platforms encoding patient-specific mutations alongside shared antigens have elicited polyfunctional CD8⁺ and CD4⁺ T-cell responses in melanoma, particularly when administered with anti–PD–1 therapy, and have been associated with improved recurrence-free outcomes in high-risk settings [68, 81]. These findings support the premise that combining antigen classes can strengthen immune activation while maintaining manufacturability and compatibility with checkpoint inhibition.

Hybrid approaches are also motivated by biological considerations. Tumors evolve under immune pressure, and targeting a single antigen class may allow selective outgrowth of antigen-negative clones. Incorporating mutation-derived epitopes together with recurrent or pathway-associated antigens increases epitope diversity and promotes recruitment of a broader T-cell repertoire. Preclinical evidence indicates that multi-antigen mRNA constructs can achieve more durable tumor control than single-target vaccines, consistent with reduced vulnerability to immune editing [82].

Beyond classical mutation- and overexpression-derived targets, hybrid platforms can incorporate unconventional tumor-specific epitopes identified through immunopeptidomics and transcriptomic profiling [40–42, 83]. Fusion-derived neoantigens and other non-canonical peptides expand the available antigen pool and may be encoded within modular mRNA architectures without requiring structural redesign of the delivery platform [6]. The flexibility of mRNA constructs permits rapid adaptation of epitope composition while preserving standardized manufacturing workflows.

A further advantage of hybrid vaccination is dynamic adaptability. Serial tumor sequencing or ctDNA monitoring enables real-time assessment of clonal evolution, allowing refinement of antigen selection during therapy [84, 85]. This adaptive framework aligns with the iterative nature of cancer progression and supports the concept of vaccines that evolve in parallel with tumor biology.

Collectively, hybrid neoantigen vaccination represents a convergence of individualized and shared strategies. By integrating multiple antigen sources within a modular mRNA backbone, these platforms seek to balance personalization, immune breadth, and clinical practicality. As sequencing pipelines, antigen prioritization algorithms, and manufacturing timelines continue to improve, hybrid designs may offer a pragmatic pathway toward scalable precision immunotherapy.

### Off-the-shelf neo-antigen mRNA vaccination

Off-the-shelf mRNA vaccines represent the most scalable extension of neoantigen immunotherapy, prioritizing standardized manufacturing and rapid deployment over individualized tailoring [86]. While shared neoantigen strategies focus on recurrent driver mutations within defined patient subsets, off-the-shelf platforms are designed for immediate clinical availability, often stratified by common oncogenic alterations and HLA distribution patterns across populations.

Recurrent mutations such as KRAS codon 12 variants and TERT promoter alterations are particularly attractive in this context due to their prevalence across colorectal, pancreatic, lung, and glioblastoma tumors [76]. Unlike individualized constructs requiring sequential sequencing and custom synthesis, these vaccines can be produced in bulk, stored, and administered without delay [20]. The feasibility of industrial-scale deployment is supported by infrastructure established for pandemic mRNA vaccine production, including advances in formulation, cold-chain optimization, and regulatory standardization. Efforts to improve storage stability—such as spray-dried and thermostable formulations capable of room-temperature preservation and mucosal delivery—further enhance accessibility, particularly in resource-limited settings [87].

Early clinical investigations provide proof of immunogenicity. Off-the-shelf constructs such as GRT-C901/GRT-R902 have demonstrated acceptable safety profiles and measurable immune activation, including induction of CD8⁺ T-cell responses and reductions in ctDNA in subsets of patients with metastatic solid tumors [36, 37]. Although durable tumor regressions remain limited in early studies, these data suggest that standardized neoantigen platforms can generate biologically meaningful immune responses outside a personalized framework.

However, this approach faces distinct biological constraints. HLA polymorphism can restrict epitope presentation, limiting universal applicability, and tumor heterogeneity may lead to variable antigen expression across patients. These factors can attenuate T-cell priming and reduce therapeutic consistency. To address these limitations, current development efforts emphasize multi-epitope payloads incorporating frequently occurring driver mutations, stratified by population-level HLA frequencies to maximize coverage [88]. Combination with checkpoint blockade and other immune-modulating strategies may further enhance response durability.

In aggregate, off-the-shelf neoantigen vaccination represents a population-oriented strategy that leverages standardized production and rapid availability [89]. While immunogenetic variability remains a challenge, continued refinement of antigen selection, formulation stability, and combination paradigms may enable broader and more equitable access to mRNA-based cancer immunotherapy [90].

The clinical utility of different neoantigen vaccine formats may depend on disease context and tumor burden. Personalized neoantigen vaccines are most suitable for the adjuvant or minimal residual disease setting, where tumor burden is low and sufficient time exists for individualized antigen discovery and vaccine manufacturing. In contrast, shared or off-the-shelf vaccines may be more appropriate in advanced or metastatic disease, where rapid treatment initiation is required and recurrent driver mutations can be targeted across patients. Hybrid vaccine approaches, combining TSAs with shared antigens, may provide a balanced strategy that maintains specificity while enabling faster deployment [91]. Such context-dependent selection of vaccine strategies may improve clinical translation by aligning antigen design with tumor biology, treatment timing, and immune responsiveness. Biomarkers such as TMB, HLA genotype, and baseline immune infiltration may further guide the selection of the most appropriate vaccine strategy for individual patients [92].

Although multiple neoantigen vaccine platforms have been explored, including peptide-based vaccines, DNA vaccines, and viral vector approaches, mRNA vaccines offer several advantages that make them particularly attractive for personalized cancer immunotherapy. Unlike peptide vaccines, which are restricted by HLA binding and often require complex adjuvant formulations, mRNA vaccines enable endogenous antigen production within host cells and support presentation through both MHC class I and class II pathways. Compared with DNA vaccines or viral vectors, mRNA platforms avoid risks of genomic integration and allow rapid, cell-free manufacturing through in vitro transcription, enabling faster adaptation to patient-specific mutational profiles. These features have positioned mRNA vaccines as one of the most flexible and scalable platforms for neoantigen-based cancer immunotherapy [93, 94].

## Molecular and structural optimization of neoantigen mRNA vaccines

The biological properties of tumor antigens strongly influence downstream engineering decisions in mRNA vaccine design. For example, TSAs, which are often present at low abundance but exhibit minimal central tolerance, typically require sequence optimization strategies that maximize translation efficiency and ensure robust antigen presentation. In contrast, TAAs carry a higher risk of immune tolerance and therefore often require designs that promote sustained antigen expression and enhanced CD4⁺ T-cell help. Antigen clonality and expression heterogeneity also influence construct architecture, favoring multi-epitope arrays or hybrid antigen designs to broaden immune coverage. These biological considerations directly inform decisions regarding mRNA sequence engineering, UTR design, structural organization of encoded epitopes, and the choice of delivery platforms discussed here.

### Sequence engineering

**Fig. 4:**
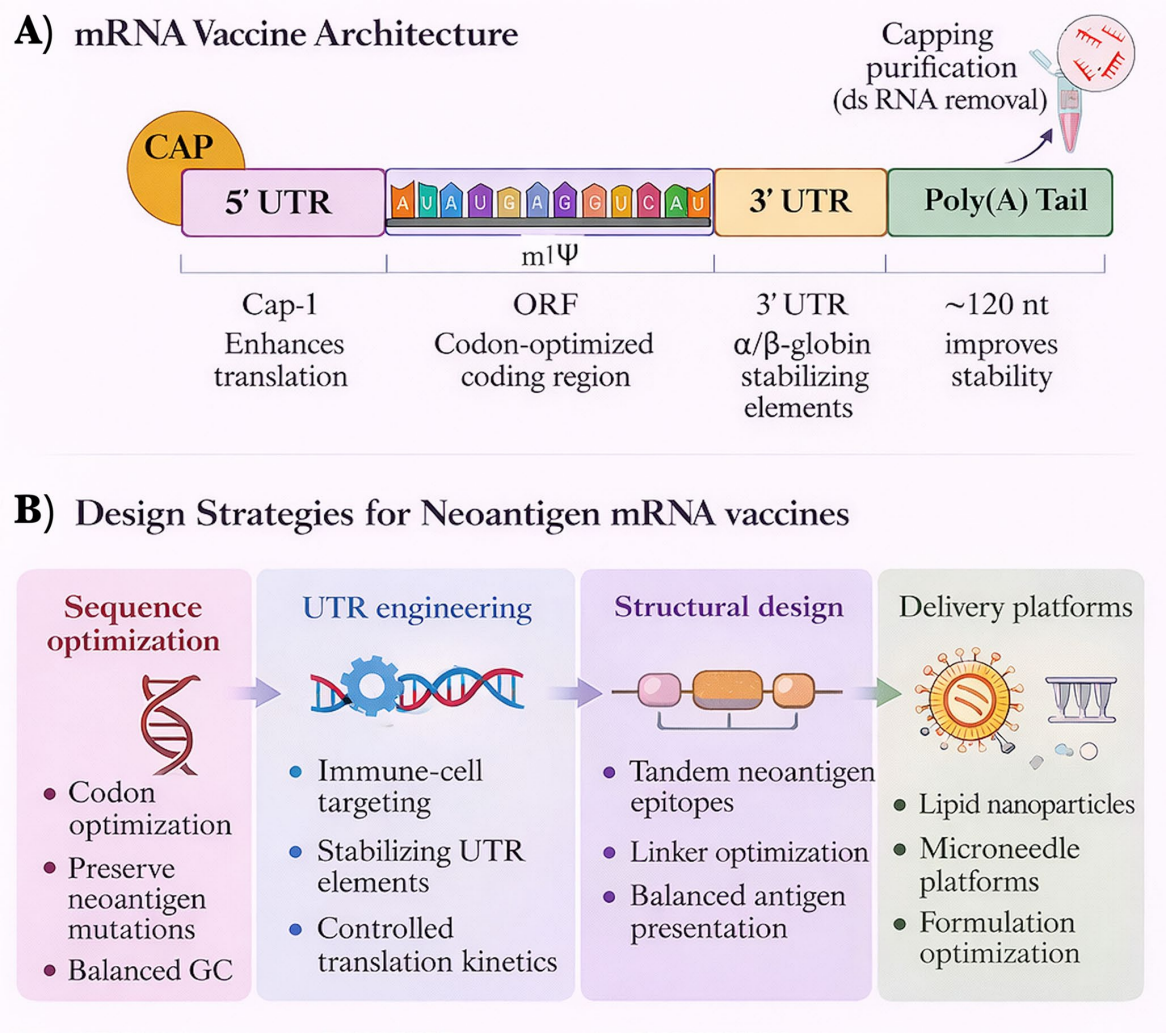
Key design principles for optimizing neoantigen mRNA vaccines. (**A**) Schematic architecture of an mRNA vaccine construct showing the 5′ cap, 5′ untranslated region (UTR), codon-optimized open reading frame (ORF) containing nucleotide modifications (e.g., m¹Ψ), 3′ UTR stabilizing elements, and poly(A) tail. Proper capping and purification remove double-stranded RNA to improve stability, translation efficiency, and safety. (**B**) Major design strategies used to optimize neoantigen mRNA vaccines include sequence optimization to preserve mutation-containing epitopes, UTR engineering to enhance mRNA stability and translation, structural design of tandem neoantigen epitopes, and advanced delivery platforms such as lipid nanoparticles and microneedle systems. Optimizing mRNA structure, antigen design, and delivery systems is critical for maximizing antigen expression and effective anti-tumor immune responses. Abbreviations: UTR, untranslated region; ORF, open reading frame; dsRNA, double-stranded RNA; LNP, lipid nanoparticle

The development of effective neoantigen mRNA vaccines begins with sequence engineering, which tailors the encoded transcript to the biological characteristics of each antigen class. TSAs, TAAs, hybrid constructs, and shared or off-the-shelf antigens differ substantially in expression level, immunogenic potential, and immune tolerance risk. These differences influence codon optimization strategies, nucleotide modification choices, and regulatory sequence design [95]. Accordingly, sequence engineering aims to maximize antigen expression and immunogenicity while maintaining transcript stability and avoiding excessive innate immune activation [96] (Fig. 4).

**Fig. 5:**
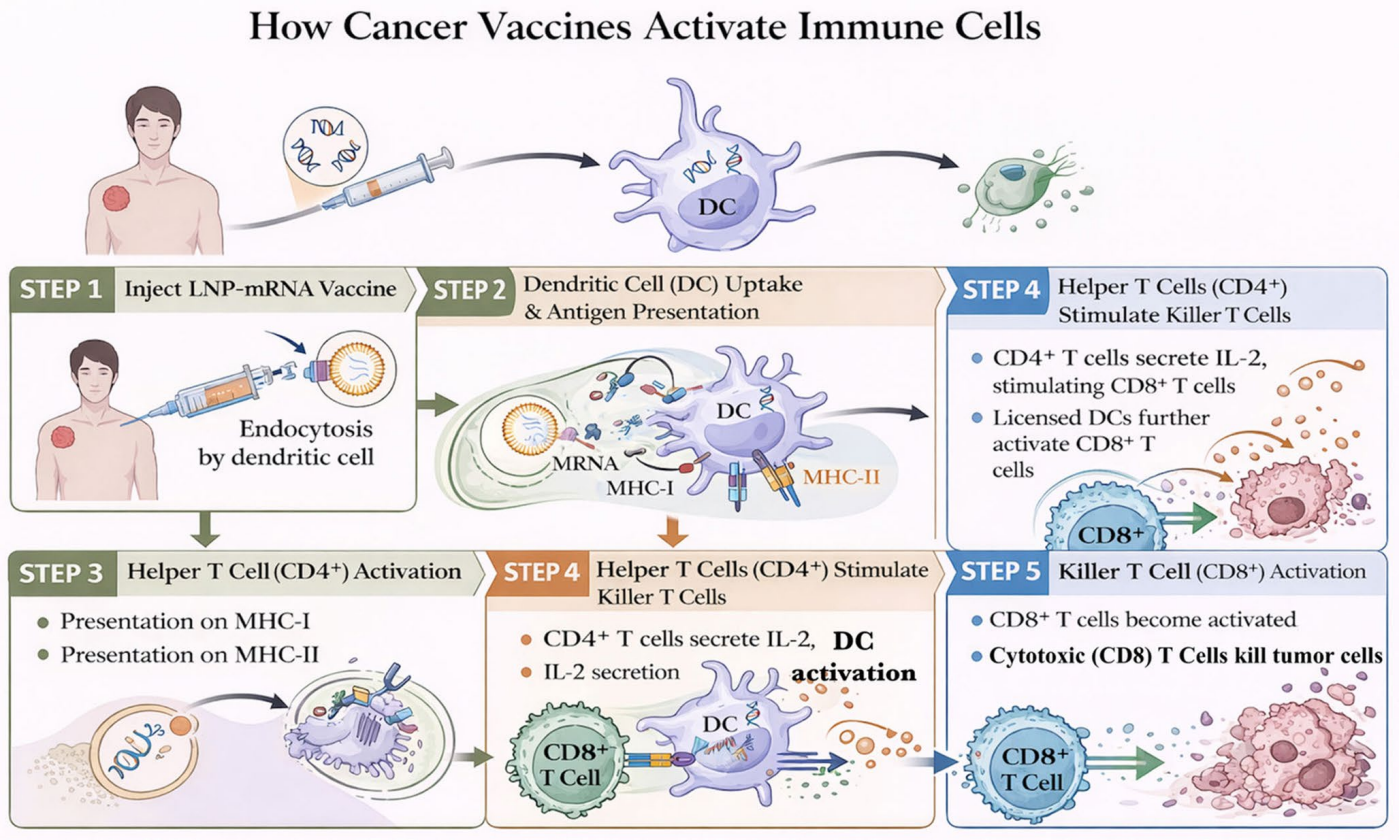
Mechanism of immune activation by mRNA cancer vaccines. After injection, lipid nanoparticle (LNP)–encapsulated mRNA vaccines are taken up by dendritic cells through endocytosis. The delivered mRNA is translated into antigenic peptides that are processed and presented on MHC class I and II molecules. Antigen presentation activates CD4⁺ helper T cells and CD8⁺ cytotoxic T cells, while CD4⁺ T cells further enhance CD8⁺ responses through cytokine secretion and dendritic cell licensing. Activated CD8⁺ T cells then recognize and kill tumor cells expressing the corresponding antigens. mRNA vaccines stimulate coordinated innate and adaptive immune responses that generate tumor-specific cytotoxic T-cell activity. Abbreviations: APCs, antigen-presenting cells; DCs, dendritic cells

For TSAs, the primary objective is to preserve mutation-containing epitopes while ensuring efficient protein translation. Because many neoantigens arise from single nucleotide variants embedded within otherwise normal protein sequences, codon optimization must maintain the mutated residue and surrounding epitope context while adjusting other codons to enhance translational efficiency. Patient-specific optimization pipelines increasingly incorporate machine-learning–guided algorithms that preserve mutation-adjacent amino acid motifs while modifying synonymous codons to improve ribosome processivity and protein output [97, 98].

In contrast, TAA constructs often encode longer or full-length proteins, such as WT1 or MUC1, which may be susceptible to extensive RNA secondary structure formation. To mitigate this effect, sequence designs frequently incorporate moderate GC content (approximately 45–50%), which reduces stable RNA folding and improves ribosomal accessibility, thereby enhancing translation efficiency in antigen-presenting cells [99].

In addition to codon selection, nucleoside modification is a central component of sequence engineering. Modified nucleotides can improve transcript stability, enhance translational output, and modulate innate immune sensing. Among these, N1-methyl-pseudouridine (m¹Ψ) is widely used in experimental mRNA vaccine platforms because it reduces recognition by innate immune receptors such as Toll-like receptors while maintaining high translational efficiency. This modification has been incorporated in several neoantigen vaccine platforms, including shared or off-the-shelf constructs targeting driver mutations such as KRAS^G12D^ [100].

Different antigen classes may require distinct nucleoside modification strategies. In many TAA-based constructs, pseudouridine or 5-methoxyuridine is used to maintain efficient translation while preserving a moderate level of immune stimulation that supports antigen presentation without provoking excessive inflammatory signaling. Hybrid vaccine constructs, which combine TSAs and TAAs within a single transcript, sometimes employ tiered modification strategies in which m¹Ψ is preferentially applied to TSA regions to maximize translation efficiency, while alternative modifications such as pseudouridine are used in TAA segments to maintain balanced immune activation [101].

Overall, sequence engineering provides the first level of immunoengineering control in neoantigen mRNA vaccine design. By integrating codon optimization, nucleotide modification, and antigen-specific sequence constraints, these strategies ensure that encoded antigens are expressed efficiently while preserving the mutation-specific epitopes required for effective anti-tumor immune recognition.

### UTR engineering and translational control

The untranslated regions (UTRs) of mRNA transcripts regulate translation by controlling ribosome recruitment, transcript stability, and interactions with RNA-binding proteins and microRNAs. The 5′ UTR primarily influences translation initiation through its secondary structure and Kozak consensus sequence, whereas the 3′ UTR modulates transcript stability and translational efficiency via binding factors such as HuR and poly(A)-binding protein (PABP) (Fig. 4) [102, 103]. In mRNA vaccine platforms, rational UTR engineering enables precise control of antigen expression kinetics while minimizing excessive innate immune activation.

Several studies have shown that UTRs derived from dendritic cell–associated transcripts can enhance translation within antigen-presenting cells (APCs). For example, regulatory regions from genes such as CD40 or LAMP3 can recruit cell-specific RNA-binding proteins that promote efficient translation in dendritic cells, thereby improving antigen presentation without altering the delivery tropism of the LNP carrier [104, 105].

UTR design is often adapted to the immunological properties of the encoded antigen. TSA constructs typically encode low-abundance mutation-derived epitopes that require efficient translation to achieve sufficient antigen presentation [106]. To support rapid protein synthesis, UTRs derived from immune-response transcripts such as IFN-β or IL-12 are sometimes incorporated because these regulatory regions promote strong translation in activated dendritic cells—the principal APCs responsible for priming naïve CD8⁺ T cells [107]. These UTRs enhance antigen expression in immune-active tissues, particularly lymph nodes where dendritic cell density and antigen-processing capacity are highest [108]. In addition, synthetic 5′ UTRs optimized for rapid ribosome scanning are frequently combined with relatively short-lived 3′ UTR elements to achieve strong but transient antigen expression. This strategy promotes efficient cytotoxic T-cell priming while limiting prolonged cytokine production that could contribute to immune exhaustion [109].

In contrast, TAA constructs often aim to sustain antigen expression to support efficient MHC class II presentation and CD4⁺ T-cell activation, which are essential for overcoming immune tolerance and reinforcing adaptive immunity. To achieve this effect, stabilizing UTRs derived from transcripts such as β-globin or GM-CSF are frequently incorporated to extend mRNA half-life and maintain translation within professional APCs including dendritic cells and macrophages. Longer-lived 3′ UTR elements prolong antigen persistence and enhance T-cell help and antibody responses [110]. For example, incorporation of β-globin UTRs has been reported to increase transcript stability by more than 2.5-fold in dendritic cells compared with certain viral-derived regulatory regions.

Thus, the distinction between TSA and TAA UTR designs primarily reflects differences in desired immune kinetics rather than differences in APC targeting. TSA constructs typically favor short-term, high-intensity antigen expression to rapidly activate CD8⁺ cytotoxic responses, whereas TAA constructs emphasize sustained translation to reinforce CD4⁺ helper T-cell activity and immune support [54].

Hybrid vaccine platforms introduce additional design complexity because they must balance these distinct antigen expression profiles within a single construct. In some strategies, differential UTR engineering is used to modulate antigen kinetics—for example, pairing shorter-lived regulatory elements such as truncated α-globin 3′ UTRs with TSA sequences while stabilizing TAA regions with longer β-globin UTR variants [111]. This asynchronous expression strategy can mimic physiological immune activation patterns, supporting coordinated CD8⁺ and CD4⁺ T-cell responses while reducing the risk of clonal anergy.

For shared or off-the-shelf neoantigen vaccine platforms, which rely on fixed antigen sequences intended for broad patient populations, UTR design must support consistent translation across diverse cell types. In these systems, consensus-engineered regulatory regions derived from housekeeping genes such as α-tubulin or eIF4G are often employed because they produce balanced translational activity in multiple cell types [112]. Combining optimized 5′ UTR Kozak sequences with GC-balanced 3′ UTR elements further stabilizes translation efficiency and reduces variability across patients.

Overall, UTR engineering represents a central component of mRNA vaccine optimization. By modulating translation kinetics and transcript stability, UTR design enables antigen-specific tuning of immune activation: TSA constructs prioritize rapid and transient expression to stimulate cytotoxic responses, whereas TAA constructs emphasize sustained antigen availability to reinforce helper T-cell activity. Hybrid and shared vaccine strategies integrate these approaches to achieve physiologically balanced antigen presentation and broad immunogenic coverage.Advances in RNA structure modeling and codon optimization further refine these design strategies. The folding pattern of an RNA transcript can influence ribosome accessibility and transcript stability, thereby shaping translational efficiency and antigen production [97]. As computational tools for RNA structure prediction improve, these approaches are increasingly integrated into the rational design of next-generation mRNA vaccine constructs.

### Structural architecture and antigen processing

Structural design refers to the internal organization of the mRNA sequence—including coding region arrangement, epitope order, linker composition, and ORF structure—rather than the three-dimensional conformation of the RNA molecule. These architectural features determine how encoded antigens are processed intracellularly and ultimately presented through MHC pathways [95]. Consequently, structural layout must be tailored to the immunological objective of the vaccine platform.

For TSA vaccines, tandem minigene constructs are commonly used to encode multiple patient-specific mutations within a single transcript. These minigenes are typically separated by short synthetic linkers to maintain balanced proteasomal processing and prevent epitope dominance that could restrict T-cell repertoire diversity [113]. Proper epitope spacing helps ensure that multiple neoepitopes are processed efficiently and presented across diverse human leukocyte antigen (HLA) alleles.

To enhance antigen expression levels, some platforms employ self-amplifying RNA (saRNA) constructs incorporating alphavirus-derived replicase elements. These systems amplify the RNA template intracellularly, increasing antigen production by one to two orders of magnitude while preserving the transient and non-integrating nature of mRNA vaccines [114]. Higher antigen expression can enhance immune priming without requiring higher vaccine doses.

Structural features can also direct antigen processing toward specific immune pathways. Incorporation of proteasomal targeting motifs, such as ubiquitin tags or degron sequences, promotes rapid cytosolic degradation and improves peptide generation for MHC class I presentation, thereby strengthening CD8⁺ cytotoxic T-cell responses. In contrast, antigens intended to stimulate CD4⁺ T-cell responses are often fused to lysosomal-targeting domains such as lysosome-associated membrane protein 1 (LAMP1), which redirects proteins toward the endosomal–lysosomal pathway and enhances MHC class II presentation [67, 115, 116]. The cytoplasmic tail of LAMP1 contains a tyrosine-based sorting motif (YXXΦ) that interacts with adaptor protein complexes (AP-1 and AP-3), guiding the fusion protein from the Golgi apparatus to late endosomes and lysosomes where antigen processing occurs. In preclinical models, LAMP1–antigen fusion constructs significantly increased CD4⁺ T-cell proliferation and cytokine production compared with cytosolic antigen expression [116].

Hybrid vaccine constructs present additional structural complexity. In these systems, TSAs and TAAs may be encoded within separate expression modules using polycistronic architectures or internal ribosome entry site (IRES) elements. Alternatively, 2 A peptide linkers can be used to produce multiple antigen products from a single transcript [117]. Maintaining appropriate epitope spacing is essential to prevent proteasomal interference and preserve efficient antigen processing. Experimental studies using hybrid constructs in glioma models demonstrated that separating TSA and TAA epitopes into distinct expression modules enhanced both CD8⁺ and CD4⁺ immune responses by more than 50% [118].

Shared neoantigen or off-the-shelf platforms also rely on multi-epitope arrays incorporating recurrent driver mutations or conserved tumor antigens. These sequences must be carefully arranged to ensure efficient antigen processing and broad HLA coverage across diverse patient populations [119]. Synthetic linkers such as AAY or GPGPG are frequently used to separate epitopes and minimize structural interference during proteasomal cleavage [120].

In some cases, structural design is complemented by heterologous prime–boost strategies, where an initial viral-vector priming vaccination is followed by mRNA boosting to reinforce recall responses and improve T-cell durability, as explored in clinical trials such as Gritstone’s adenoviral–mRNA platform (NCT03639714) [36, 37, 121]. Across these diverse approaches, effective structural architecture must preserve physiological antigen processing while minimizing immunodominance and maximizing the breadth of tumor-specific T-cell responses.

### Delivery platforms and formulation logic

Efficient delivery systems are a central determinant of the clinical performance of neoantigen mRNA vaccines because they influence antigen biodistribution, cellular uptake, immune activation, toxicity, and manufacturing scalability [122]. Delivery design becomes particularly important when targeting TSAs, which are highly individualized and typically expressed at low levels. In these cases, effective immune priming requires precise delivery of the mRNA payload to APCs, especially dendritic cells that initiate T-cell responses [123, 124]. LNP systems have therefore become the dominant delivery platform for mRNA vaccines because they protect RNA from degradation while enabling efficient cellular uptake and endosomal release.

To improve immunological specificity, LNPs are increasingly engineered with targeting elements that promote uptake by dendritic cells. Surface ligands such as mannose or antibodies directed against receptors like DEC205 can guide nanoparticles toward APC populations in lymphoid tissues [125]. In experimental TSA vaccine models, mannose-modified LNPs have been shown to enhance accumulation in lymph nodes and improve antigen-specific CD8⁺ T-cell responses [126]. Some formulations also incorporate innate immune stimulators such as STING or RIG-I agonists within the same nanoparticle, enabling localized activation of antiviral signaling pathways that amplify T-cell priming and enhance neoantigen immunogenicity [127].

For TAAs or shared driver mutations such as KRAS^G12D^ and TP53^R175H^, broader delivery strategies may be preferable. PEGylated LNPs and cationic nanoemulsions can distribute more widely across tissues and are often combined with immune-stimulating adjuvants, including TLR3 or TLR7/8 agonists, to help overcome immune tolerance mechanisms [128]. Alternative delivery technologies are also being explored. Intradermal electroporation and microneedle array systems, for example, enable direct targeting of skin-resident antigen-presenting cells. These approaches may be particularly useful for hybrid vaccine designs that combine TSAs and TAAs and therefore require coordinated activation of multiple immune pathways.

Another emerging strategy involves sequential or layered delivery approaches. In this framework, an initial priming dose is designed to reach lymph node–resident APCs, followed by a booster administration that promotes broader antigen exposure throughout the immune system. Such spatial and temporal coordination of antigen presentation can increase the magnitude and durability of vaccine-induced T-cell responses [129].

In parallel with advances in targeting strategies, improvements in ionizable lipid chemistry have significantly expanded the capabilities of LNP delivery systems. One recent approach introduced a modular “plug-and-play” platform for synthesizing biodegradable ionizable lipids, which form the structural core of LNPs. Using a simple combinatorial reaction scheme, researchers generated large libraries of candidate lipids that could be rapidly screened for efficient mRNA delivery [130]. From these libraries, several formulations demonstrated improved delivery efficiency compared with earlier benchmark lipids used in mRNA therapeutics [131]. These modular lipid systems are designed to enhance endosomal escape and improve intracellular RNA release while maintaining low toxicity.

Additional innovations have focused on achieving organ-specific delivery. Siloxane-incorporated lipid nanoparticles (SiLNPs) represent one such development. By introducing siloxane groups into ionizable lipid structures, researchers generated a range of nanoparticle formulations with distinct tissue distribution patterns [132]. Some variants preferentially delivered mRNA to liver tissue, whereas others demonstrated enhanced delivery to pulmonary endothelial cells or immune cells in the spleen. These findings suggest that modifications to lipid structure can influence tissue targeting and may enable more precise delivery of mRNA therapeutics to specific organs or immune cell populations. Improved membrane interaction and endosomal escape properties are thought to contribute to the enhanced delivery efficiency observed in these systems.

Together, these advances considerably expand the delivery toolkit available for mRNA-based cancer immunotherapies. Modern LNP platforms can now be engineered to optimize biodistribution, enhance immune activation, and support tissue-specific targeting, enabling delivery strategies tailored to different antigen classes and therapeutic goals [132].

Finally, practical considerations such as manufacturing scalability and global distribution are becoming increasingly important for the development of neoantigen vaccines. Technologies that reduce dependence on ultra-cold storage conditions are therefore receiving growing attention. Lyophilized or spray-dried LNP formulations, as well as self-amplifying RNA platforms capable of maintaining stability at moderate temperatures, offer promising approaches for improving storage and transport logistics [133]. Recent initiatives have also explored dry powder formulations for mucosal delivery of mRNA vaccines, further expanding the potential routes of administration and simplifying vaccine deployment [134].

The relative advantages of these emerging delivery systems, including modular ionizable lipids, siloxane-based nanoparticles, targeted surface modifications, and thermostable formulations, are summarized in Table 2. Together, these technologies provide a framework for selecting delivery strategies that align with the immunological requirements, antigen types, and clinical deployment scenarios of neoantigen-based cancer vaccines.

**Table 2:** Comparison of two representative mRNA vaccine delivery platforms: lipid nanoparticle (LNP)-based intramuscular delivery and spray-dried mucosal formulations. The table summarizes key parameters including stability, targeting capacity, immunogenicity, clinical development status, dosing route, patient preference, scalability, and platform limitations

Parameter	LNP-based intramuscular delivery	Spray-dried mucosal delivery
Stability	Requires − 20 °C–− 80 °C; stable ≤ 6 months; cold-chain dependent	Room-temperature stable ≥ 6 weeks; no cold-chain; stable post-reconstitution
Targeting	Ligand-conjugated LNPs enhance tumor/lymph node uptake (5–10× over naked mRNA)	M-cell targeting ligands enhance nasal mucosal uptake (3–5× vs. soluble protein)
Immunogenicity	Induces systemic CD8⁺ T cells; Th1-biased cytokines; dose-sparing possible	Strong mucosal IgA + systemic CD4⁺/CD8⁺ T cell activation; local tolerance reduced
Clinical Status	BNT122, mRNA-4157 in Phase II/III; well-characterized safety/tolerability	CEPI/NIH-backed Phase I trials (e.g., Ethris); high mucosal immunogenicity shown
Dosing Route	Intramuscular injection; requires trained personnel	Nasal spray/powder inhaler; self-administered or low-skill deployment
Patient Preference	~ 78% accept IM with pre-vaccine counseling (adults, cancer trials)	> 90% prefer nasal route in pediatric/needle-phobic cohorts
Scalability	Established for GMP; cold chain increases cost/logistics burden	Low-cost, ambient storage; ideal for LMICs and decentralized settings
Key Limitations	Cold-chain requirements; lipid-related reactogenicity; anti-PEG antibodies with repeated dosing; systemic biodistribution.	Nasal clearance and mucus barrier reduce delivery efficiency; variability in patient dosing; fewer clinical validation studies.

Abbreviations: LNP, lipid nanoparticle; mRNA, messenger RNA; IgA, immunoglobulin A; CD4⁺/CD8⁺, cluster of differentiation 4 and 8 T lymphocytes; Th1, T helper 1; IM, intramuscular; GMP, good manufacturing practice; LMICs, low- and middle-income countries; PEG, polyethylene glycol; CEPI, Coalition for Epidemic Preparedness Innovations; NIH, National Institutes of Health

## Functional immunogenic optimization and clinical positioning

The clinical success of neoantigen mRNA vaccines will depend not only on antigen discovery or molecular engineering but on functional integration within complex immune and clinical landscapes. While early-phase trials demonstrate immunogenicity, translating immune activation into durable tumor control remains contingent upon several biological and logistical constraints.

Biomarkers are increasingly important for determining which vaccine strategy is most appropriate for individual patients. Tumors with high tumor mutational burden (TMB) typically generate larger repertoires of tumor-specific neoantigens and may therefore benefit more from fully personalized TSA vaccines [135]. In contrast, cancers with lower mutational burden but recurrent driver mutations may be better suited to shared or off-the-shelf neoantigen vaccines targeting alterations such as KRAS or TERT promoter mutations [136]. HLA genotype further influences epitope presentation and determines whether specific neoantigens can be efficiently recognized by cytotoxic T cells [137]. In addition, biomarkers reflecting immune context—such as baseline immune infiltration, interferon signaling signatures, or ctDNA dynamics—may help distinguish potential responders from non-responders and guide the selection of combination therapies that enhance vaccine efficacy [138, 139]. Integrating genomic and immunological biomarkers may help guide the selection of appropriate neoantigen vaccine strategies across different patient populations. Key biomarker–strategy relationships are summarized in Table 3.

**Table 3:** Biomarker-guided selection of neoantigen mRNA vaccine strategies. This table summarizes how key genomic and immunological biomarkers may help guide the selection of appropriate neoantigen vaccine strategies across different tumor contexts

Biomarker context	Biological implication	Vaccine strategy
High TMB tumors	Many private neoantigens	Personalized TSA mRNA vaccine
Low TMB with driver mutations	Few private, shared oncogenic mutations	Shared/off-the-shelf neoantigen vaccine
Favorable HLA genotype	Efficient antigen presentation	Personalized TSA or TSA/TAA hybrid
Immune-cold TME	Low T-cell infiltration	Vaccine + immune modulators
ctDNA-positive MRD state	Residual disease after therapy	Adjuvant personalized vaccine

Abbreviations: TMB, tumor mutational burden; TSA, tumor-specific antigen; TAA, tumor-associated antigen; HLA, human leukocyte antigen; TME, tumor microenvironment; ctDNA, circulating tumor DNA; MRD, minimal residual disease; ICI, immune checkpoint inhibitor

A central challenge lies in calibrating innate immune activation. Effective vaccination requires sufficient dendritic cell licensing to support robust CD8⁺ and CD4⁺ T-cell priming. LNP formulations contribute intrinsic adjuvant effects through activation of pathways such as TLR4 and NF-κB, and additional immune-stimulatory motifs may further amplify Type I interferon signaling [140–142]. However, excessive innate activation can impair mRNA translation, induce systemic inflammatory toxicity, and potentially accelerate T-cell exhaustion. The therapeutic window between productive immune priming and detrimental hyperinflammation remains incompletely defined in humans, particularly in patients with prior checkpoint exposure or chronic inflammatory states. Immunogenic optimization, therefore, must prioritize controlled activation rather than maximal stimulation.

Another important safety consideration relates to the biodistribution of lipid nanoparticle–encapsulated mRNA after systemic administration [143]. Although dendritic cells represent the primary target for antigen presentation, multiple cell types—including hepatocytes, endothelial cells, and tissue-resident immune cells—may transiently take up exogenous mRNA. This raises the theoretical possibility of off-target antigen expression or unintended immune activation in non-malignant tissues [144]. Current vaccine designs mitigate this risk through optimized UTR, rapid mRNA degradation kinetics, and dose-controlled delivery systems, but continued monitoring of biodistribution and safety remains essential in clinical development [145].

Tumor biology presents an equally formidable barrier. Intratumoral heterogeneity, clonal evolution under immune pressure, and antigen loss can limit the durability of neoantigen-targeted responses. Even highly immunogenic epitopes may be downregulated or edited over time. These constraints suggest that neoantigen mRNA vaccines may achieve their greatest impact in settings of minimal residual disease or early-stage intervention, where tumor burden is low and immune suppression less entrenched. In advanced or immune-excluded malignancies, vaccination alone is unlikely to suffice [146]. Accordingly, combination strategies are emerging as a functional necessity rather than an adjunctive enhancement. Checkpoint blockade can release vaccine-expanded T-cell clones from exhaustion [147]., while focal radiotherapy or selected chemotherapeutics may increase antigen presentation and transiently inflame the tumor microenvironment [148]. Oncolytic viral priming and STING pathway activation further illustrate the concept of conditioning the tumor bed before or alongside systemic vaccination [149–151]. In this framework, the mRNA vaccine acts as a systemic immune primer integrated within a spatially coordinated therapeutic sequence. The optimal ordering, dosing, and patient selection criteria for these combinations remain to be defined in prospective trials.

Adaptive personalization represents another frontier. ctDNA monitoring enables detection of emergent resistance mutations and dynamic assessment of clonal architecture. In principle, these data streams could inform iterative redesign of vaccine payloads during active treatment. Yet the feasibility of repeated sequencing, redesign, manufacturing, and regulatory oversight within clinically actionable timelines remains an open question. Automation of IVT optimization and closed-system synthesis platforms suggests that turnaround times can be shortened [152–155], but scalable implementation will require regulatory models that validate manufacturing platforms rather than individual sequences [156, 157].

Vaccine platforms are increasingly being explored in combination with adoptive cellular therapies such as chimeric antigen receptor T (CAR-T) cells. While CAR-T therapy has produced major clinical successes in hematological malignancies, its application in solid tumors remains limited by insufficient TSAs, poor T-cell infiltration, and an immunosuppressive tumor microenvironment. Vaccine-based strategies may help address these challenges by enhancing antigen-specific immune priming and supporting CAR-T cell expansion and persistence. Various vaccine modalities, including mRNA, peptide, viral vector, and dendritic cell platforms, are under investigation, with early studies suggesting that mRNA vaccines may improve CAR-T cell activity and durability in solid tumors [158].

Finally, equitable deployment will depend on resolving manufacturing scalability and cost constraints. While personalized platforms push the boundaries of precision oncology, shared and off-the-shelf strategies offer greater standardization and rapid availability. The long-term trajectory of neoantigen vaccination will likely involve coexistence of these approaches, with clinical positioning guided by tumor mutational burden, HLA profile, disease stage, and resource context. Thermostable formulations and distributed production systems may expand accessibility, but global integration will require coordinated regulatory adaptation and sustained investment [159–162].

Different neoantigen vaccine strategies may also be suited to distinct clinical contexts. Personalized vaccines are most rational in the adjuvant or minimal residual disease setting, where time for antigen discovery exists and tumor burden is low. Shared or off-the-shelf vaccines may be more appropriate for advanced or rapidly progressing cancers, where rapid treatment initiation is essential. Hybrid approaches may bridge these contexts by combining broadly shared antigens with patient-specific mutations to balance speed and specificity [27, 32].

Collectively, functional immunogenic optimization must be understood as systems integration rather than incremental refinement. Antigen selection, transcript engineering, delivery chemistry, immune conditioning, manufacturing logistics, and clinical context are interdependent variables. Progress will depend on defining where neoantigen mRNA vaccines provide durable benefit, identifying the patients most likely to respond, and aligning technological sophistication with translational feasibility.

## Future directions and clinical translation

**Fig. 6:**
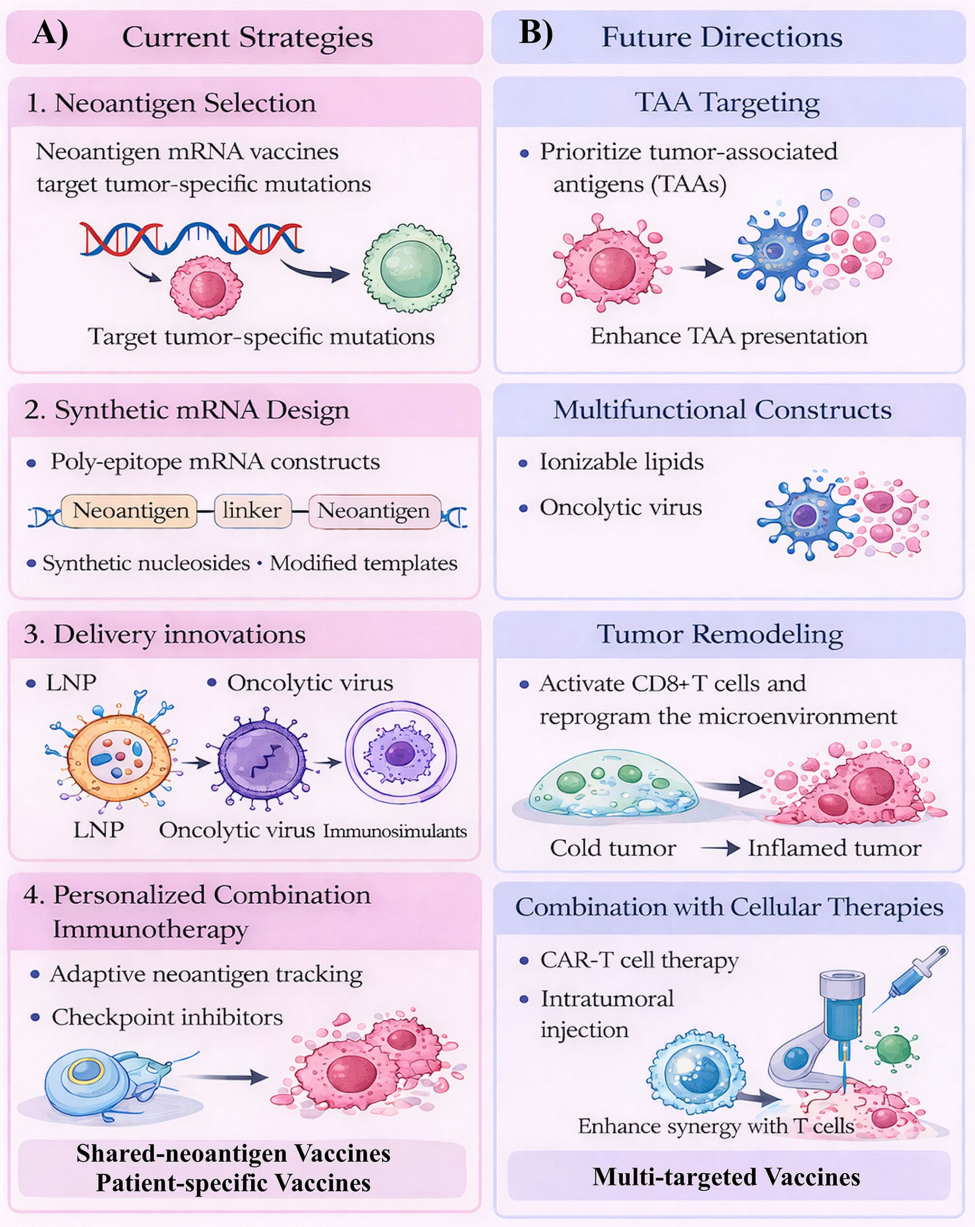
Current strategies and future directions for neoantigen mRNA cancer vaccines. This schematic compares established strategies used in current neoantigen mRNA vaccine development with emerging approaches aimed at improving clinical efficacy. **(A)** Current strategies include optimized neoantigen selection, synthetic mRNA design, advanced delivery platforms such as lipid nanoparticles, and personalized combination immunotherapy. **(B)** Future directions focus on expanding target antigens, developing multifunctional vaccine constructs, remodeling the tumor microenvironment to enhance immune infiltration, and integrating mRNA vaccines with cellular therapies and multi-targeted immunotherapies. Next-generation neoantigen mRNA vaccines will likely combine improved antigen design, smarter delivery systems, and synergistic immunotherapies to overcome tumor heterogeneity and enhance anti-tumor immunity

Neoantigen mRNA vaccines are transitioning from conceptual frameworks to structured clinical programs, yet their long-term impact will depend on how effectively biological sophistication aligns with clinical pragmatism (Fig. 6). The next phase of development will likely be defined not by incremental improvements in antigen prediction but by integration of adaptive immunization, immune-environment remodeling, scalable manufacturing, and regulatory modernization.

A major frontier is dynamic personalization. Advances in ctDNA monitoring now permit detection of emergent resistance mutations at very low variant allele frequencies, enabling near–real-time mapping of clonal evolution. Integrating these data streams into modular vaccine platforms raises the possibility of iterative antigen updating during treatment. Such adaptive vaccination may be particularly relevant in tumors characterized by rapid immune-driven antigen drift, including melanoma and NSCLC [163]. However, the feasibility of repeatedly redesigning and manufacturing personalized constructs within clinically meaningful timeframes remains to be demonstrated outside controlled trial settings.

These design choices also influence regulatory evaluation. Trials incorporating biomarker-based patient stratification and surrogate endpoints such as ctDNA clearance or neoantigen-specific T-cell expansion may provide earlier signals of vaccine activity, which can accelerate regulatory review in early-phase studies. Adaptive trial designs may therefore play an important role in demonstrating clinical benefit for personalized mRNA vaccine platforms [164, 165].

Equally important is the challenge of immune exclusion. Many solid tumors, including pancreatic cancer, gliomas, and TNBC, exhibit limited T-cell infiltration and dominant immunosuppressive signaling. In these contexts, antigen delivery alone may be insufficient. Combination strategies involving checkpoint blockade, metabolic modulation, focal radiotherapy, or oncolytic viral priming are being explored to convert non-inflamed tumors into vaccine-permissive environments [149–151, 166–168]. The emerging paradigm suggests that mRNA vaccines function most effectively as immune primers within a deliberately conditioned tumor microenvironment rather than as standalone agents. Determining the optimal sequencing and dosing of such combinations remains an active area of investigation.

Manufacturing scalability represents another decisive factor. Automation of IVT optimization and closed-system mRNA synthesis platforms demonstrate that personalized production can be streamlined [152–155]. Although decentralized GMP microfactories and platform-level regulatory approval represent promising directions for accelerating personalized mRNA vaccine production, these models remain largely aspirational. Current neoantigen vaccine pipelines typically rely on centralized manufacturing systems due to the need for controlled quality testing, individualized sequence validation, and rapid batch production timelines. Clinical studies of personalized RNA vaccines have demonstrated that the design, manufacturing, and release of individualized vaccine batches still require coordinated centralized workflows and strict quality control processes [15, 94]. In addition, broader analyses of neoantigen vaccine development highlight regulatory and manufacturing challenges associated with individualized biologics, including product comparability, scalability, and standardized quality control frameworks [17, 93].

Finally, global accessibility will test whether neoantigen vaccination becomes a niche precision therapy or a broadly deployable modality. Thermostable formulations and cold-chain–independent LNP formats expand the logistical horizon [159], but equitable access will depend on international collaboration, cost reduction, and robust data governance to protect patient genomic information [160, 161]. As shared neoantigen strategies mature, population-level targeting of recurrent mutations prevalent in high-incidence cancers may offer an intermediate path between personalization and global scalability [162].

Collectively, the future of neoantigen mRNA vaccines lies in convergence. Antigen discovery, molecular engineering, immune modulation, automated manufacturing, and regulatory innovation must evolve in parallel. The field is moving beyond proof-of-concept immunogenicity toward defining where and in whom these vaccines provide durable clinical benefit. Whether in adjuvant settings, minimal residual disease, or combination regimens designed to overcome immune exclusion, the next decade will determine whether neoantigen vaccination becomes a foundational pillar of precision oncology or remains confined to highly selected patient subsets.

## Concluding remarks

Neoantigen mRNA vaccination has evolved from an experimental concept into a structured clinical strategy grounded in tumor genomics, immune engineering, and modular manufacturing. Across individualized, shared, hybrid, and off-the-shelf platforms, the central principle remains consistent: encoding tumor-restricted antigens to elicit targeted T-cell responses while minimizing off-target toxicity.

Clinical studies, particularly in melanoma and non-small cell lung cancer, demonstrate that neoantigen vaccination can expand tumor-reactive T-cell clones and improve recurrence-free outcomes when integrated with checkpoint blockade [68, 147]. These data support the concept that mRNA vaccines function most effectively as immune primers within combination regimens rather than as standalone interventions. At the same time, biological challenges—including tumor heterogeneity, immune editing, HLA diversity, and variability in innate activation thresholds—underscore that immunogenicity alone does not guarantee durable clinical benefit.

Technological advances in transcript engineering, delivery systems, and automated manufacturing have improved reproducibility and reduced turnaround times, bringing personalized platforms closer to practical implementation [152–154]. However, scalable deployment will depend on regulatory adaptation toward platform-based validation and on defining clear clinical contexts in which vaccination provides additive benefit. Current evidence suggests that adjuvant and minimal residual disease settings may offer the most favorable therapeutic window.

Ultimately, the trajectory of neoantigen mRNA vaccines will be determined by integration rather than innovation alone. Antigen selection, immune modulation, manufacturing logistics, and patient stratification must operate within a coherent clinical framework. As combination strategies mature and adaptive personalization becomes feasible, neoantigen vaccination may transition from proof-of-concept immunogenicity to a durable component of precision oncology. Whether it becomes broadly deployable or remains confined to selected indications will depend on resolving the biological and regulatory challenges outlined in this review.

Despite significant advances in antigen discovery, mRNA engineering, and delivery platforms, several challenges remain, including tumor heterogeneity, HLA restriction, immune exclusion in solid tumors, and the logistical complexity of rapid personalized vaccine manufacturing. Furthermore, regulatory frameworks remain a significant bottleneck. Current approval pathways at agencies such as the FDA and EMA are largely designed for fixed biologic products, whereas personalized mRNA vaccines involve rapidly changing sequences and individualized manufacturing. As a result, regulatory agencies are beginning to explore platform-based evaluation approaches, although clear guidelines for decentralized production systems are still under development.

## Data Availability

No datasets were generated or analysed during the current study.

## Abbreviations

mRNA: Messenger RNA
INT: Individualized neoantigen therapy
TSA: Tumor-specific antigen
TAA: Tumor-associated antigen
WES: Whole-exome sequencing
SNV: Single-nucleotide variant
indels: Insertions and deletions
PDAC: Pancreatic ductal adenocarcinoma
GMP: Good Manufacturing Practice
lncRNAs: Long noncoding RNAs
ctDNA: Circulating tumor DNA
CEA: Carcinoembryonic antigen
WT1: Wilms’ tumor protein 1
MUC1: Mucin-1
TMVs: Tumor membrane vesicles
TNBC: Triple-negative breast cancer
Tregs: Regulatory T cells
MDSCs: Myeloid-derived suppressor cells
saRNA: Self-amplifying RNA
IRES: Internal ribosome entry site
ILs: Ionizable lipids
SiLNPs: Siloxane-incorporated nanoparticles
APCs: Antigen-presenting cells
UTRs: Untranslated regions
